# The veiled right kidney sign

**DOI:** 10.4103/0971-4065.43697

**Published:** 2008-07

**Authors:** A. Y. Lakshmi, B. Vijaya Lakshmi, S. Sarala, B. Mutteswaraiah

**Affiliations:** Department of Radiology and Imaging, Sri Venkateswara Institute of Medical Sciences, Tirupati, Andhra Pradesh - 517 507, India; 1 Department of General Surgery, Sri Venkateswara Institute of Medical Sciences, Tirupati, Andhra Pradesh - 517 507, India

**Fig. 1:**
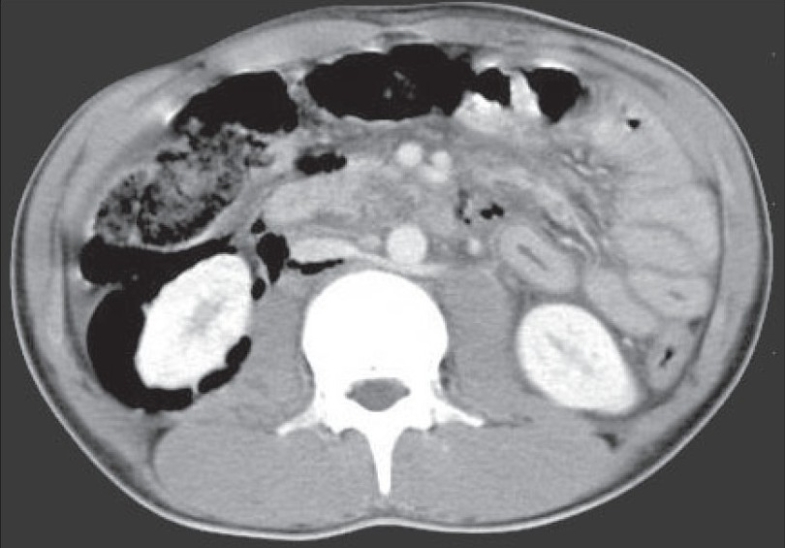
Contrast-enhanced computed tomography axial section at the level of the lower pole of the kidneys showing evidence of foci of air in the mesentery and air collection around the right kidney

The “veiled right kidney sign” is an uncommon entity in which the right kidney is masked by the collection of air around it in the retroperitoneum.^1^,^2^ A 20 year-old male presented with vomiting and distension of the abdomen following an accidental fall on a rock, sustaining a blunt injury over his abdomen. Hematological and routine blood biochemistry tests did not reveal any abnormalities. A plain skiagram of the abdomen (an anteroposterior (AP) view) showed the presence of air in the right perinephric region masking the right kidney. Contrast computed tomography of the abdomen revealed the presence of air in the right perinephric space and a moderate amount of fluid in the peritoneum [Fig. 1]. Laporotomy showed an injury in the third part of the duodenum. Closure of the duodenal laceration, gastrojejunostomy, and a decompression tube duodenostomy were performed after which the patient was discharged on recovery.

Traumatic blunt duodenal injury is associated with about 12% mortality. Morbidity and mortality from duodenal wounds may be reduced by early hospitalization, early diagnosis, and consequently, earlier surgical repair.^3^ The presence of air in the right perinephric region—“the veiled right kidney sign” —helps in this situation as an invaluable pointer towards appropriate management.^1^,^2^
